# Institutional Needs

**Published:** 1920-01-24

**Authors:** 


### Institutional Needs.

### "ENOLIN" TOOTH PASTE.

(MESSRS. A. AND F. CARRERAS, 207 KING STREET, HAMMERSMITH, W.)

JUST as British dental surgery has amply shown during the past twenty years that it is no longer, as for a time in 'nineties of last century it was, behind the times as compared with American science, so too (but more recently) have various British manufacturers fully demonstrated that their toilet wares are at the least equal to any of the imported dentifrices, etc., which have had so great a vogue since large sections of the public became convinced of the importance of attention to the teeth. Prominent among those responsible for this welcome alteration of a regrettable state of affairs is the firm of Carreras, manufacturers of the "Enolin" tooth paste and of other well-known specialities. It is claimed on behalf of "Enolin," and we believe justly, that its antiseptic and anti-bacterial properties are unsurpassed, though admittedly no dentifrice can ever completely sterilise the mouth and gums; that it contains nothing in the way of gritty particles such as can injure the enamel of the teeth; and that it is pleasant and stimulating to use. The manufacturers send out this extremely valuable preparation in two strengths, "mild" and "strong." Having tested both, the writer casts his vote in favour of the latter; but he has habituated himself for years to rather stiff brushes and to tooth pastes having some "bite" in them, so possibly to many other people of more susceptible mucous membranes the milder variety might seem preferable. At all events, he has no hesitation in recommending both sorts to those in search of a pleasing and efficient tooth paste of British origin.

### IGLODINE.

(THE IGLODINE CO., LTD., NEWCASTLE-ON-TYNE.)

THIS antiseptic is a pure drug—*i.e.*, a definite chemical compound (tri-iodo-ethyl-phenyl, $C_4H_7I_3$), not a mixture of materials—*i.e.*, a proprietary patent medicine; and in consequence can be, and is, sold without a revenue stamp on the package. From the information submitted to us, duly authenticated, it seems that iglodine has been on the market for many years, but it is only lately that its full capabilities have been appreciated, at any rate in the South of England. Iglodine went on war service in France, Italy, Mesopotamia, and elsewhere, which doubtless accounts for the growth of its recognition and popularity. The bacteriological evidence of its efficacy as a bactericide is extraordinarily favourable; a solution of a strength of 1 in 500 in water is the strength most commonly used clinically, with results as satisfactory as those of the laboratory. Iglodine has but little odour, and that not unpleasant; it is not irritant, and can indeed be taken internally. Lastly, iglodine is economical; it is sold at 18s. the gallon, or made up as an ointment at 4s. 6d. per lb. It is especially recommended as an antiseptic in first-aid work, for which it is extensively used in the North of England. The makers supply first-aid cabinets and equipment, complying with the Home Office regulations, in which iglodine holds a prominent place. Hospital casualty rooms and operating theatres could do worse than make a trial of this valuable antiseptic.

